# Coordinated Response to SARS, Vancouver, Canada

**DOI:** 10.3201/eid1201.050327

**Published:** 2006-01

**Authors:** Danuta M. Skowronski, Martin Petric, Patricia Daly, Robert A. Parker, Elizabeth Bryce, Patrick W. Doyle, Michael A. Noble, Diane L. Roscoe, Joan Tomblin, Tung C. Yang, Mel Krajden, David M. Patrick, Babak Pourbohloul, Swee Han Goh, William R. Bowie, Tim F. Booth, S. Aleina Tweed, Thomas L. Perry, Allison McGeer, Robert C. Brunham

**Affiliations:** * British Columbia Centre for Disease Control, Vancouver, British Columbia, Canada;; † Vancouver Coastal Health, Vancouver, British Columbia, Canada;; ‡ Fraser Health, New Westminster, British Columbia, Canada;; § Royal Columbian Hospital, New Westminster, British Columbia, Canada;; ¶ St. Paul's Hospital, Vancouver, British Columbia, Canada;; # University of British Columbia, Vancouver, British Columbia, Canada;; ** National Microbiology Laboratory, Winnipeg, Manitoba, Canada;; †† Mount Sinai Hospital, Toronto, Ontario, Canada

**Keywords:** SARS, emerging pathogens, outbreak, nosocomial infections, coronavirus, dispatch

## Abstract

Two Canadian urban areas received travelers with severe acute respiratory syndrome (SARS) before the World Health Organization issued its alert. By July 2003, Vancouver had identified 5 cases (4 imported); Toronto reported 247 cases (3 imported) and 43 deaths. Baseline preparedness for pandemic threats may account for the absence of sustained transmission and fewer cases of SARS in Vancouver.

In Canada, 2 urban areas received returning travelers infected with severe acute respiratory syndrome–associated coronavirus (SARS-CoV) from the original Hotel M cluster in Hong Kong. These travelers returned to Canada before the World Health Organization (WHO) issued its first global alert on March 12, 2003. One infected traveler from Hotel M returned to the greater Toronto area (GTA, population 4.7 million), Ontario; 2 returned to the Vancouver census metropolitan area (VCMA, population 2.0 million), British Columbia (BC). GTA, Ontario, is located in central Canada ≈4,000 km from VCMA, BC, which is the westernmost province of Canada. Control of SARS in both GTA and VCMA was by a national, publicly funded, but provincially administered healthcare system. Whereas GTA experienced sustained transmission, VCMA did not. Ultimately, GTA reported 247 patients with SARS and 43 related deaths; 3 cases were imported. VCMA identified 5 confirmed cases, 4 of which were imported (1–5). The experience with SARS in Vancouver highlights how a well-coordinated response of baseline precautions, reinforced through timely public health alerts and periodic infection control audits, can mitigate outbreaks due to emerging respiratory-borne pathogens.

## The Outbreak

Neutralization antibody titers to SARS-CoV among patients in VCMA are shown in Table 1. SARS-CoV was also confirmed in all but patient 1 by reverse transcription–polymerase chain reaction with multiple distinct primer sets applied to multiple specimens (3,6–9).

**Table 1:** SARS CoV antibody titers by microneutralization assay in persons with laboratory-confirmed SARS in VCMA*

Serum sample no.	Patient 0		Patient 1		Patient 2		Patient 3		Patient 4	
	Days after onset	Titer	Days after onset	Titer	Days after onset	Titer	Days after onset	Titer	Days after onset	Titer
1	10	1:32	16	1:128	4	<1:8	3	<1:8	11	<1:8
2	19	1:128	29	1:32	14	1:32	36	1:128	28	1:32
3	45	1:32	637	1:64	20	1:128	224	1:128	203	1:128
4					217	1:128	466	1:64	463	1:64
5					481	1:64				

*By number of days after symptom onset that serum was collected. SARS, severe acute respiratory syndrome; CoV, coronavirus; VCMA, Vancouver census metropolitan area.

Patient 0 and patient 1 were a couple, who stayed on the 14th floor of Hotel M from February 20 to 24, 2003, and again from March 3 to 6. Both became ill on February 26 (Table 2). They returned to Canada on March 6 and went directly from the airport to their physician in Vancouver on March 7 (day 9 of illness). The husband (patient 0) was sent directly to the emergency room of a tertiary-care hospital (hospital A), arriving at 1:55 p.m. Within 15 minutes, full respiratory precautions were instituted. He was moved to a private room in the emergency room at 2:20 p.m. and transferred to a negative-pressure isolation room (NPIR) at 4:20 p.m. He was admitted into an NPIR of the intensive care unit (ICU) with full respiratory precautions at 6 a.m. on March 8 (Table 2).

**Table 2:** Epidemiologic and clinical profile of patients with confirmed SARS, Vancouver*

Patient characteristics			Patient 0	Patient 1	Patient 2	Patient 3	Patient 4
Baseline characteristics							
		Sex	Male	Female	Female	Male	Female
		Age (y)	55	54	64	49	44
	Medical condition		No	Diabetes	Hypertension	No	No
Epidemiologic characteristics							
		Travel related	Yes	Yes	Yes	Yes	No
		City of likely source of SARS	Hong Kong	Hong Kong	Hong Kong	Hong Kong	VCMA
		Known contact with SARS	No	No	Yes	No	Yes
		Likely date(s) of exposure, 2003	Feb 21	Feb 21	Mar 19	Mar 28–30	Mar 29 or Mar 30
		Likely setting of exposure	Hotel M	Hotel M	Dinner party	Amoy Gardens	Hospital B
		Date of return to Canada, 2003	Mar 6	Mar 6	Mar 20	Mar 30	NA
Clinical profile							
Symptoms and onset, 2003							
		Malaise	Feb 26	Feb 26	Mar 24	No	Apr 4
		Myalgia	No	Feb 28	Mar 24	Apr 1	Apr 10
		Headache	Feb 28	Feb 28	Mar 27	Apr 1	Apr 4
		Fever	Feb 28	Feb. 28	Mar 29	Apr 1	Apr 15
		Chills	Feb 28	Feb 28	Mar 29	No	No
		Chest discomfort	No	No	Mar 24	No	No
		Cough	Mar 1	No	Mar 29	No	Apr 11
		Shortness of breath	Mar 1	No	Mar 29	Apr 3	Apr 11
		Nausea	No	No	Mar 27	No	No
		Vomiting	No	No	No	No	No
		Diarrhea	Mar 7	No	Mar 28	Apr 6	Apr 11
Hospitalized			Yes	No	Yes	Yes	Yes
Oxygen saturation (%) on room air at admission			45	NA	80	97; fell to 62 within 3 h	86
Aerosolized medication or nebulizer before isolation			No	No	No	No	No
Date of hospital admission			Mar 7	NA	Mar 28	Apr 3	Apr 15
No. days after symptom onset that patient was hospitalized			10		4	2	11
Date of final hospital discharge			Jun 12	NA	Apr 21	Apr 21	May 24
ICU			Yes	No	Yes	No	Yes
Date of ICU admission			Mar 8	NA	Apr 1	NA	Apr 15
Date of ICU discharge			May 13	NA	Apr 18	NA	Apr 24
Mechanical ventilation			Yes	No	Yes	No	No
Delay to implementation at hospital of:							
Respiratory precautions†			15 min‡	NA	Immediate§	Immediate§	7 min¶
Negative-pressure isolation			165 min‡	NA	Immediate§	Immediate§	11 min¶

*SARS, severe acute respiratory syndrome; VCMA, Vancouver census metropolitan area; NA, not applicable; ICU, intensive care unit; ER, emergency room; NPIR, negative-pressure isolation room. †Defined as standard precautions (gloves, gown, eyewear) plus N95 mask and mask on patient when transported. Full respiratory precautions also include NPIR. ‡Arrived in triage March 7, 2003 1:55 p.m. By 2:10 p.m., admission sheet advises "full respiratory precautions" be taken. By 2:20 p.m. in single room in ER. Transferred to NPIR in ER at 4:40 p.m. §Arrived at hospital masked and admitted directly into NPIR. ¶Arrived in ER on April 14, 2003, at 9:49 p.m. Identified as suspected SARS patient at 9:56 p.m. Masked and transferred to NPIR in ER at 10 p.m. Admitted to ICU NPIR on April 15.

His wife, patient 1, was recovering from mild illness, and no further follow-up was arranged. The couple had no other household contacts. Review confirmed that symptoms had not developed in any of the 148 hospital workers involved in patient 0's care by 10 days after his arrival at the hospital. The family physician had no detectable neutralizing antibody to SARS-CoV when tested at day 496.

Patient 2 of the VCMA had prolonged contact abroad with 2 family members in Hong Kong, who subsequently died from SARS. Although asymptomatic, she went to her physician in VCMA on March 26 because she was concerned about her exposure. Chest radiograph showed bilateral consolidation, and she was directed, masked, to hospital B, where she was admitted directly to an NPIR. She was transferred to the ICU of hospital C for assisted ventilation (Table 2). Neither of her 2 household contacts had detectable SARS-CoV antibody at day 215.

Patient 3 stayed at Amoy Gardens March 28–30 (10). Upon return, he remained self-isolated in the VCMA in the basement suite of his home with no contacts (household members were nevertheless quarantined, but they remained asymptomatic). Masked and short of breath, he sought treatment at hospital A on April 3. Initial chest radiograph was normal, but computed tomography scan showed widespread, patchy, ground-glass opacification of both lungs. He was admitted to hospital D directly to an NPIR (Table 2). His son, who drove him to hospital masked, had no detectable antibody to SARS-CoV at day 200.

Patient 4 of the VCMA was a nurse who cared for patient 2 at hospital B from March 29 to 30. At the time, patient 2 was receiving oxygen by mask and nebulization therapy. Patient 4 assisted patient 2 in using the toilet, which was flushed with lid raised in her presence. She followed guidelines in place at the time, but these did not include eye protection. Symptoms developed in the nurse on April 4. She went to hospital E on April 15, where she was admitted directly to an NPIR. Her only household contact remained asymptomatic. Neither he, nor a physician who examined her on April 11, had detectable SARS CoV antibody at 200 and 365 days, respectively.

All 5 patients with SARS in VCMA recovered fully. No additional unrecognized spread was evident. None of 442 staff members of hospitals A–E who participated in a voluntary serosurvey had detectable SARS-CoV antibody by microneutralization assay (details available from corresponding author, upon request).

## Conclusions

Mathematical models for SARS, incorporating contact network theory, stress the importance of patient 0 in predicting the likelihood of an epidemic (11). This likelihood can be determined by the transmissibility of the agent, number of contacts of patient 0, and number of persons infected between patient 0 (the first patient infected) and intervention on the index patient (the first recognized case-patient). From this perspective, the circumstances of patient 0 in Vancouver compared to patient 0 in the Toronto, Canada, outbreak, merit closer examination.

Approximately 2,000 passengers land in Vancouver on direct flights from Hong Kong and mainland China every day compared with 500 on average to Toronto. As such, Vancouver is a potential gateway to North America for emerging pathogens from Asia. Because of this perceived risk, the BC Centre for Disease Control (BCCDC) had been increasing preparedness for pandemic threats for several years. An electronic distribution system was established to regularly disseminate communicable disease bulletins to healthcare facilities across the province. When a cluster of unexplained atypical pneumonia in China was reported almost simultaneously with reemergence of influenza A H5N1 in Hong Kong, BCCDC used this well-established communication network to issue an alert on February 20, 2003. The alert requested enhanced vigilance for severe influenzalike illness in returning travelers from mainland China or Hong Kong or among their close contacts. Alerts were repeated February 24, February 28, and March 12, 2003. Before patient 0's arrival, the emergency room at hospital A also participated in an infection control audit that emphasized that barrier precautions should be applied with all acute-onset respiratory infections. Patient 0 thus became the index patient in VCMA and was managed cautiously, even before WHO special alerts were issued. He sought treatment at the cusp of his peak infectious period at a tertiary-care hospital that had been repeatedly primed towards precaution. As a returned traveler, he was a first-generation case. He had no family contacts other than his wife, with whom he had traveled. Infection control precautions were implemented almost immediately upon his arrival at the hospital, limiting opportunities for spread.

When SARS arose in Ontario, a comparable agency to BCCDC did not exist. Responsibility for communicable disease control had shifted over the course of several years to local health boards, which created a decentralized system (12). Patient 0 in Toronto also stayed at Hotel M with her spouse from February 18 to 21. She returned to the GTA on February 23 to an apartment she shared with 5 family members (5,13). She died at home on March 5. During this period, she infected her 43-year-old son. This son became Toronto's index patient, a locally acquired, second-generation case (5,13). He went to a community hospital on March 7, the same day as Vancouver's patient 0, but was not recognized as a special threat. He was placed in general observation in the emergency room, where he remained for 18 hours and where he was given nebulized salbutamol. He was not placed in airborne isolation until he had been at the hospital for 21 hours; droplet and contact precautions were later begun on March 10 (5,13). By the time WHO issued its global alert, at least 14 persons in GTA had already become infected through 4 generations of spread: half within patient 0's family and the remainder among healthcare contacts. Concern about severe illness in family members as they sought treatment at the hospital prompted an evening phone consultation on March 13 from an infection control practitioner in Toronto to the BCCDC in Vancouver. This call linked the separate Toronto and Vancouver cases to events in Asia and led to recognition that SARS had spread beyond that region. It also prompted WHO to issue a rare travel advisory on March 15 (14). Thereafter, awareness of precautions to be taken was enhanced everywhere, and further importations into Canada (Vancouver and Toronto) did not result in spread.

Ultimately, standard droplet and contact precautions proved an effective barrier to SARS except in the context of superspreading events such as aerosolizing procedures (3). Low inherent transmissibility, combined with the delay in peak infectivity until well into the course of serious illness, may explain why SARS was primarily a nosocomial infection and why so few countries experienced outbreaks (3). Patient 0 tests the baseline capacity of a system to respond to emerging threats before they are known or recognized. While favorable random chance may have played a role, Vancouver's response to SARS should not be dismissed on the basis of luck alone. Pasteur's edict that "chance favors only the prepared mind" may have modern relevance to the prepared healthcare system (15). The response to patient 0 in Vancouver highlights the importance of central coordination, baseline preparedness at the local level, and an efficient network of communication in mitigating outbreaks. Baseline preparedness should include barrier precautions in the care of all acute-onset respiratory infections. These should be reinforced through timely public health alerts and periodic infection control audits.
